# Mania a Potu

**Published:** 1845-01

**Authors:** 


*Mania a Potu.*—The diminution in the number of cases of *mania a potu* (in the Philadelphia Hospital) is striking, in the first six years there being two hundred and sixty-four deaths, while in the second half of the series there are but forty-nine. The striking disproportion is probably to be accounted for by the change in habits induced by the progression of temperance. The admissions now are by no means as frequent as formerly, and very rarely advance to the third stage of this disease.

On the authority of Dr. Gerhard, I may state that the treatment in the men's department, until the year 1837, had been by opiates, and that the number of deaths to those admitted, had been in the proportion of 1 to 10. He then tried stimulation by alcohol, and we have this result:

| | Admitted. | Cured. | Died. |
|---|---|---|---|
| Intoxication, | 61 | 61 | 0 |
| Delirium Tremens, 1st stage, | 87 | 87 | 0 |
| " " 2d " | 73 | 73 | 0 |
| " " 3d " | 2 | 1 | 1 |

"The fatal case died a few hours after his entrance, and had been treated by opium in the city." But that this stimulant is not required, I give the report of Dr. Dunglison, who has charge of the Women's Asylum for six months of the year. He "used not a drop of alcoholic liquor." His course "has been entirely eclectic, in many cases expectant," and in its support we have the following:

| | Admitted. | Cured. | Died. |
|---|---|---|---|
| Intoxication, | 44 | 44 | 0 |
| Delirium Tremens, 1st stage, | 55 | 55 | 0 |
| " " 2d " | 19 | 19 | 0 |
| " " 3d " | 10 | 9 | 1 |

"which last died on the morning after admission, and had been treated elsewhere for nearly a week."

*Am. Jour. Med. Sci.*, for Octo er.

